# Subjective cognitive decline and subsequent dementia: a nationwide cohort study of 579,710 people aged 66 years in South Korea

**DOI:** 10.1186/s13195-020-00618-1

**Published:** 2020-05-06

**Authors:** Yeong Chan Lee, Jae Myeong Kang, Hyewon Lee, Kiwon Kim, Soyeon Kim, Tae Yang Yu, Eun-Mi Lee, Clara Tammy Kim, Doh Kwan Kim, Matthew Lewis, Hong-Hee Won, Frank Jessen, Woojae Myung

**Affiliations:** 1 Department of Neuropsychiatry, Seoul National University Bundang Hospital Hospital, 29 Gumi-ro 173 Beon-gil, Bundang-gu, Seongnam-si, 13619 Gyeonggi-do Republic of Korea; 2 Samsung Advanced Institute for Health Sciences and Technology (SAIHST), Sungkyunkwan University, Samsung Medical Center, 81 Irwon-ro, Gangnam-gu, Seoul, 06351 Republic of Korea; 3 Department of Psychiatry, Gil Medical Center, Gachon University College of Medicine, Incheon, Republic of Korea; 4 Institute of Health and Environment, Seoul National University, Seoul, Republic of Korea; 5 Department of Psychiatry, Veteran Health Service Medical Center, Seoul, Republic of Korea; 6 Division of Endocrinology and Metabolism, Department of Medicine, Wonkwang Medical Center, Wonkwang University School of Medicine, Iksan, Republic of Korea; 7 Department of Health Science, Dongduk Women’s University, Seoul, Republic of Korea; 8 Institute of Life and Death Studies, Hallym University, Chuncheon, Gangwon-do Republic of Korea; 9 Department of Psychiatry, Samsung Medical Center, Sungkyunkwan University School of Medicine, Seoul, Republic of Korea; 10 The Department of General Practice, Melbourne Medical School, The University of Melbourne, Melbourne, Victoria Australia; 11 Department of Psychiatry, University of Cologne, Cologne, Germany

**Keywords:** Subjective cognitive decline, Subjective memory impairment, Alzheimer’s disease, Dementia, Depression, Preclinical Alzheimer’s disease

## Abstract

**Background:**

Subjective cognitive decline (SCD) is a potential risk factor for dementia. We aimed to investigate the association between SCD and subsequent dementia in a nationwide population-based cohort in South Korea.

**Methods:**

This cohort included 579,710 66-year-old adults who were followed for a total of 3,870,293 person-years (average 6.68 ± 1.33 years per person). All subjects completed a questionnaire about subjective memory impairment, the Pre-screening Korean Dementia Screening Questionnaire (KDSQ-P), which included a validated 5-item derivative, and were determined to have SCD based on a single question assessing memory decline. Depressive symptoms were assessed in all subjects using a 3-item modified geriatric depression scale. Hazard ratios were estimated using the Cox proportional hazards model and compared between subjects with and without SCD.

**Results:**

Compared to subjects without SCD, those with SCD were more likely to develop dementia (incidence per 1000 person-years: non-SCD, 5.66; SCD, 8.59). After adjusting for potential confounding factors, the risk of subsequent dementia significantly increased in subjects with SCD, with an adjusted hazard ratio (aHR) of 1.38 (95% confidence interval [CI] 1.34 to 1.41). The risk of subsequent dementia was greatly increased in subjects with higher KDSQ-P scores (aHR = 2.77, 95% CI 2.35 to 3.27). A significant association between SCD and dementia was observed in both depressive and non-depressive symptom groups (aHR = 1.50, 95% CI 1.42 to 1.57 in subjects with depressive symptoms; aHR = 1.33, 95% CI 1.29 to 1.37 in subjects without depressive symptoms; *P* = 0.001).

**Conclusions:**

In this population of 66-year-old individuals, SCD was significantly associated with an increased risk of subsequent dementia. This association was found in both depressive and non-depressive groups, with an increased risk of dementia in the presence of depressive symptoms. Our findings suggest that SCD indicates a risk for dementia. Further studies are needed to delineate potential approaches to preventing the development of dementia in individuals with SCD.

## Background

Dementia represents one of the most prevalent neurodegenerative disorders worldwide and is present in approximately 10% of people aged 65 years and older [1]. Moreover, the public health burden of dementia is growing more rapidly than any other disease [2]. It has been reported that almost 80% of people worry about developing dementia [3] and identify dementia as their most feared illness, over cancer, heart disease, stroke, and diabetes [4]. Aging is concomitant with increased episodes of forgetfulness, and memory complaints are prevalent in approximately 25 to 50% of older adults [5]. Previous studies have found that memory complaints may relate to subclinical psychiatric symptoms [5–7], but they can also represent an early indicator of dementia, including Alzheimer’s disease (AD) [8–12]. Considering the growing number of patients with dementia and the associated medical and societal burden, it is important to characterize at-risk groups or preclinical states of dementia in order to facilitate early interventions to reduce cognitive impairment in the future.

As part of this effort, considerable progress has been made investigating the prospective dementia risk associated with subjective cognitive decline (SCD) [13, 14]. SCD refers to a subjective experience of cognitive decline without objective cognitive deficits [14]. Research indicates that SCD may represent an early symptom of AD signifying the preclinical stage [9–12], which can progress to mild cognitive impairment (MCI) and dementia in the AD continuum [12–15]. However, SCD is also associated with poor physical health and with psychiatric disorders such as depression, which confounds the association with dementia [16, 17]. For example, depression is a major risk factor for dementia, and previous work has indicated that SCD could be linked with subsyndromal depressive symptoms rather than with subsequent dementia [18]. The considerable heterogeneity present across numerous small studies has contributed to conflicting results and has prevented consensus in the field [16–18].

Regarding the recruitment setting, it has been observed that SCD in memory clinic cases increased the risk of dementia [10], whereas SCD in community populations showed less significant or non-significant associations [10, 19]. Given that the concerns and health-seeking behaviors of community-recruited older adults may differ from those of memory clinic patients [10], investigations using a large community sample would improve the accuracy for estimating the SCD-associated risk for incident dementia in the general population.

This study analyzed a nationwide population-based cohort that includes 51.8% of the 66-year-old adult population in South Korea. We aimed (1) to determine whether the risk of subsequent dementia increases in subjects with SCD compared to those without SCD, (2) to evaluate whether the severity of subjective memory impairment is associated with subsequent dementia, and (3) to examine whether depressive symptoms affect the association between SCD and subsequent dementia.

## Methods

### Data sources and study cohort

Data were obtained from the South Korean National Health Insurance Service (NHIS) database (supplementary methods) [20]. The NHIS provides mandatory healthcare for 97% of South Koreans under a single-payer model. Further, it provides the National Screening Program for Transitional Ages (NSPTA), an age-specific national health examination program for all Korean citizens aged 40 and 66 [21]. Our study population consisted of a subset of individuals from the NHIS database who participated in the NSPTA at age 66, between 2009 and 2011. The study population covered 51.8% of the total South Korean population aged 66 during the enrolment period. This study was approved by the Institutional Review Board of Seoul National University Bundang Hospital. Because the NHIS provided encrypted data to protect private information, the need to obtain informed consent was waived (approval No. X-1901-517-902).

### Inclusion and exclusion criteria

We included all subjects who had available information on the Pre-screening Korean Dementia Screening Questionnaire (KDSQ-P), a cognitive function questionnaire [22]. The exclusion criteria were as follows: (1) individuals who reported impaired function in activities of daily living (ADLs) because of possible pre-existing dementia (ADLs were assessed by a questionnaire consisting of six questions derived from a modified Korean Activities of Daily Living Scale and Korean Instrumental Activities of Daily Living Scale [23]); (2) individuals with dementia (ICD-10 [International Classification of Disease, 10th revision] code F00-F03, G30, or G31), mild cognitive impairment (ICD-10 code F06·7), or documented history of dementia medication (donepezil, rivastigmine, galantamine, or memantine) before the index date; (3) individuals with a psychotic disorder (ICD-10 code F20-F29; these criteria were applied to approximately meet the research criteria for pre-mild cognitive impairment SCD suggested in a previous study [14]); (4) individuals with missing or duplicate data on the primary variable of interest or covariates; (5) individuals with outlier values in continuous variables (mean ± 4 standard deviations); and (6) individuals who died or dropped out between the time they participated in the NSPTA and the index date. From the 650,861 subjects who took the NSPTA, 71,150 subjects (10.9%) were excluded according to the exclusion criteria (see Figure S1 in the online supplement).

### Primary independent variable of interest: SCD

SCD was defined as an answer of “yes” to item 2 of the KDSQ-P. KDSQ-P is a validated questionnaire that consists of five questions [22]. Each item can be self-answered with three possible choices: “no,” “sometimes yes,” or “frequently yes,” scored as 0, 1, and 2, respectively. Overall KDSQ-P scores range from 0 to 10, with higher scores indicating greater degrees of subjective memory impairment. Those who score ≥ 4 points are advised to seek further evaluation of their cognitive function. All items of the KDSQ-P are presented in Table S1 in the online supplement. We defined SCD based on scoring a 1 or 2 (a positive answer) on the responses to item 2, which asks about the subjective decline in the memory domain and was suggested in the conceptual framework for research on SCD: “Do you think your memory has declined compared to a year ago?” [14].

### Subjective memory impairment and depressive symptoms

The severity of the subjective memory impairment was defined using the total score on the KDSQ-P ranging from 0 to 10 [22]. The presence of depressive symptoms was defined as a Depression Screening Questionnaire (DSQ) score > 0. This questionnaire includes three questions derived from a modified geriatric depression scale [24] (e.g., “Have you lost much of your activity or motivation these days?,” “Do you feel that you are worthless now?,” and “Do you feel that you have no hope now?”). Each question can be self-answered with two possible choices, “yes” or “no,” scored as 1 or 0, respectively. Total DSQ scores range from 0 to 3, with higher scores indicating more depressive symptoms.

### Primary outcomes

The primary outcome was the incidence of dementia following SCD. Dementia was defined based on the International Statistical Classification of Diseases, 10th revision codes (F00-F03, G30, or G31; Table S2) and the use of cognitive-enhancing medications based on previous studies [25, 26]. This definition is relevant to our study considering that the Korean government covers medical expenditure for dementia based on ICD-10 codes. Additionally, clinicians are required to document ICD-10 codes for dementia as well as the results of neuropsychological tests to prescribe cognitive-enhancing medications. The date of onset of dementia was considered the first date for which patients were both diagnosed with dementia and prescribed with dementia medication.

### Covariates

We assessed demographic variables such as sex and income. Lifestyle variables such as smoking status, alcohol consumption habits, and exercise frequency were included as covariates. We further adjusted for healthcare visit frequency, laboratory test results, physical examination results, and the patient’s medical history, including information related to psychiatric disorders, neurological diseases, and other medical diseases (Table S2). To assess depressive symptoms, we used the DSQ score. Detailed information is presented in the supplementary methods.

### Statistical analysis

To investigate the association between SCD and the incidence of dementia, study participants were followed from the index date (1 January of the year after each participant participated in the NSPTA) to the date of onset of dementia, death, or the end of follow-up (31 December 2017), whichever occurred first. For all participants, between-group differences for continuous variables and categorical variables were assessed using *t* tests and chi-squared tests, respectively. A Cox proportional hazard regression analysis was conducted to determine adjusted hazard ratios (aHRs) for SCD in predicting subsequent dementia, after controlling for covariates. The effect of SCD on subsequent dementia was first analyzed in an unadjusted model and then in a sex-adjusted and three additional models adjusted for various covariates (models 1 to 3). In the secondary analysis, we used the total KDSQ-P score as an independent variable to evaluate the association between the severity of subjective memory impairment and subsequent dementia. We also calculated aHR separately for score 1 and score 2 for each item of the KDSQ-P and the aHR of score 2 compared to score 1 only in the SCD group. We used an SCD and depressive symptom (coded as dichotomous variables: 0 indicating the absence of any depressive symptoms and 1 indicating the presence of any depressive symptoms among the three DSQ items) interaction term to test the potential for an interaction effect on subsequent dementia.

The proportional hazards assumption was graphically tested and verified using the Schoenfeld residual method. No variables violated the proportional hazards assumption. Multicollinearity between all covariates was tested using a variance inflation factor (VIF), and no significant collinearity was found (VIF < 4 for all variables). After conducting a survival analysis of all participants, we performed an additional analysis by sampling the control group using the propensity score matching method based on logistic regression [27] using the *Matchit* packages in R (http://cran.r-project.org). We also performed several sensitivity analyses to confirm the robustness of the main findings. Firstly, we excluded patients who developed dementia within a year of the index date because those patients may not be incident cases. Secondly, we conducted separate analyses by dementia subtypes (AD and non-AD cases) to examine whether the association between SCD and dementia differs by dementia subtypes. Thirdly, we excluded patients with a history of psychiatric disorders, patients with a history of neurological diseases, or patients with depressive symptoms according to the DSQ (DSQ > 0), because the presence of these disorders/diseases may confound the association of SCD with dementia affecting the dementia risk. Finally, we excluded patients with a KDSQ-P score ≥ 4 (the cutoff point for further dementia screening tests [22]) to more rigorously exclude preexisting dementia cases.

Statistical analyses were conducted using two-tailed tests, a significance level of 0.05, and 95% confidence intervals (CIs). All analyses were conducted using SAS Enterprise Guide version 7.2 (SAS Institute, Inc.) and R Studio version 1.0.136 (RStudio, Inc., with packages *Survival* version 2.43-3 and *Survminer* version 0.4.3).

## Results

During the period from 2009 to 2011, a total of 650,861 individuals participated in the NSPTA and had KDSQ-P information available. Of these, we excluded 21,458 individuals who reported impaired ADL function; 12,658 individuals with dementia, MCI, or with a documented history of cognitive-enhancing medication; 18,760 individuals with missing or duplicate data; 14,315 individuals with outlier data; 2632 individuals with a psychotic disorder; and 1328 individuals who died or were lost to follow-up between their NSPTA participation date and the index date. In total, 579,710 subjects were included in the final study population for analysis, of which 222,056 (38.3%) experienced SCD (see the flowchart of study participants in Figure S1 in the online supplement). They were followed for an average of 6.68 ± 1.33 years per person and a total of 3,870,293 person-years.

### Subject characteristics

The clinical and demographic characteristics of the participants at baseline are presented in Table 1. The study population consisted of 266,311 (45.9%) men and 313,399 (54.1%) women. Compared to individuals in the non-SCD group, those with SCD tended to be women, did not smoke, consumed more alcohol, exercised more, visited healthcare facilities more frequently, had more medical or medication history, had higher cholesterol levels, and had lower fasting glucose, hemoglobin, and blood pressure.

**Table 1:** Descriptive characteristics of the study population

	Total (*n* = 579,710)	Non-SCD group (*n* = 357,654)	SCD group (*n* = 222,056)	*P* value
Sex				< 0.0001
Male	266,311 (45.9%)	173,795 (48.6%)	92,516 (41.7%)	
Female	313,399 (54.1%)	183,859 (51.4%)	129,540 (58.3%)	
Income				< 0.0001
Medicaid aid	33,023 (5.7%)	19,101 (5.3%)	13,922 (6.3%)	
Group 1 (1st to 6th ventiles)	125,064 (21.6%)	82,491 (23.1%)	42,573 (19.2%)	
Group 2 (7th to 14th ventiles)	178,625 (30.8%)	110,550 (30.9%)	68,075 (30.7%)	
Group 3 (15th to 20th ventiles)	242,998 (41.9%)	145,512 (40.7%)	97,486 (43.9%)	
Lifestyle factors				
Smoking status				< 0.0001
Never smoked	406,103 (70.1%)	250,238 (70.0%)	155,865 (70.2%)	
Ex-smoker	95,502 (16.5%)	57,647 (16.1%)	37,855 (17.0%)	
Current smoker	78,105 (13.5%)	49,769 (13.9%)	28,336 (12.8%)	
Alcohol consumption				< 0.0001
No drinking: rarely	508,547 (87.7%)	314,324 (87.9%)	194,223 (87.5%)	
Light drinking: 3–4 times per week	38,840 (6.7%)	23,954 (6.7%)	14,886 (6.7%)	
Heavy drinking: almost every day	32,323 (5.6%)	19,376 (5.4%)	12,947 (5.8%)	
Exercise frequency				< 0.0001
Exercise	327,775 (56.5%)	200,723 (56.1%)	127,052 (57.2%)	
No exercise	251,935 (43.5%)	156,931 (43.9%)	95,004 (42.8%)	
Healthcare visit frequency*				
First quartile	144,858 (25.0%)	93,542 (26.2%)	51,316 (23.1%)	
Second quartile	144,945 (25.0%)	89,583 (25.1%)	55,362 (24.9%)	
Third quartile	144,988 (25.0%)	88,208 (24.7%)	56,780 (25.6%)	
Fourth quartile	144,919 (25.0%)	86,321 (24.1%)	58,598 (26.4%)	
Past medical history				
Psychiatric disorders				
Depression	48,653 (8.4%)	26,676 (7.5%)	21,977 (9.9%)	< 0.0001
Bipolar affective disorder	1891 (0.3%)	1059 (0.3%)	832 (0.4%)	< 0.0001
Substance use disorder	3522 (0.6%)	2035 (0.6%)	1487 (0.7%)	< 0.0001
Panic disorder	2315 (0.4%)	1298 (0.4%)	1017 (0.5%)	< 0.0001
Obsessive-compulsive disorder	671 (0.1%)	370 (0.1%)	301 (0.1%)	0.001
Personality disorder	237 (0.0%)	142 (0.0%)	95 (0.0%)	0.619
Other psychiatric disorders	140,212 (24.2%)	81,764 (22.9%)	58,448 (26.3%)	< 0.0001
Neurological diseases				
Cerebrovascular disease	80,212 (13.8%)	46,895 (13.1%)	33,317 (15.0%)	< 0.0001
Epilepsy	8622 (1.5%)	4807 (1.3%)	3815 (1.7%)	< 0.0001
Migraines	43,466 (7.5%)	25,304 (7.1%)	18,162 (8.2%)	< 0.0001
Headaches	70,207 (12.1%)	40,791 (11.4%)	29,416 (13.2%)	< 0.0001
Sleep disorder	63,769 (11.0%)	36,635 (10.2%)	27,134 (12.2%)	< 0.0001
Head injury	64,698 (11.2%)	39,459 (11.0%)	25,239 (11.4%)	< 0.0001
Medical diseases				
Diabetes mellitus	154,977 (26.7%)	94,346 (26.4%)	60,631 (27.3%)	< 0.0001
Myocardial infarction	8504 (1.5%)	5245 (1.5%)	3259 (1.5%)	0.981
Congestive heart failure	31,952 (5.5%)	19,222 (5.4%)	12,730 (5.7%)	< 0.0001
Liver disease	146,020 (25.2%)	88,392 (24.7%)	57,628 (26.0%)	< 0.0001
Renal disease	5669 (1.0%)	3476 (1.0%)	2193 (1.0%)	0.564
Peptic ulcer disease	259,797 (44.8%)	155,929 (43.6%)	103,868 (46.8%)	< 0.0001
Thyroid gland disorder	40,236 (6.9%)	23,104 (6.5%)	17,132 (7.7%)	< 0.0001
Asthma	123,850 (21.4%)	74,598 (20.9%)	49,252 (22.2%)	< 0.0001
Cancer	41,290 (7.1%)	24,845 (6.9%)	16,445 (7.4%)	< 0.0001
Medication history				
HMG-CoA reductase inhibitors	128,527 (22.2%)	77,725 (21.7%)	50,802 (22.9%)	< 0.0001
Diabetes medication	84,015 (14.5%)	51,878 (14.5%)	32,137 (14.5%)	0.735
Antihypertensive medication	288,262 (49.7%)	178,093 (49.8%)	110,169 (49.6%)	0.180
Antidepressants	31,079 (5.4%)	17,216 (4.8%)	13,863 (6.2%)	< 0.0001
Benzodiazepines and sleeping pills	92,444 (15.9%)	53,108 (14.8%)	39,336 (17.7%)	< 0.0001
Antiplatelet medication	140,615 (24.3%)	86,071 (24.1%)	54,544 (24.6%)	< 0.0001
Depression Screening Questionnaire score, mean (SD)	0.34 (0.79)	0.23 (0.67)	0.53 (0.92)	< 0.0001
Laboratory findings				
Cholesterol level, mean (SD), mg/dL				
LDL cholesterol	117.47 (35.58)	117.26 (35.55)	117.81 (35.64)	< 0.0001
HDL cholesterol	53.63 (13.64)	53.57 (13.61)	53.73 (13.68)	< 0.0001
Triglycerides	134.31 (70.64)	134.80 (70.97)	133.54 (70.10)	< 0.0001
Fasting glucose	101.89 (20.96)	102.11 (21.10)	101.52 (20.73)	< 0.0001
Hemoglobin	13.59 (1.40)	13.63 (1.40)	13.54 (1.39)	< 0.0001
Physical examination findings				
Body mass index	24.29 (2.98)	24.31 (2.99)	24.25 (2.98)	< 0.0001
Systolic blood pressure	128.74 (15.43)	129.05 (15.44)	128.22 (15.41)	< 0.0001
Diastolic blood pressure	78.01 (9.73)	78.18 (9.73)	77.73 (9.73)	< 0.0001

*HDL* high-density lipoprotein, *LDL* low-density lipoprotein, *SCD* subjective cognitive decline, *SD* standard deviation

*The fourth quartile group had the highest frequency of medical visits

### Risk of subsequent dementia according to SCD

**Fig. 1:**
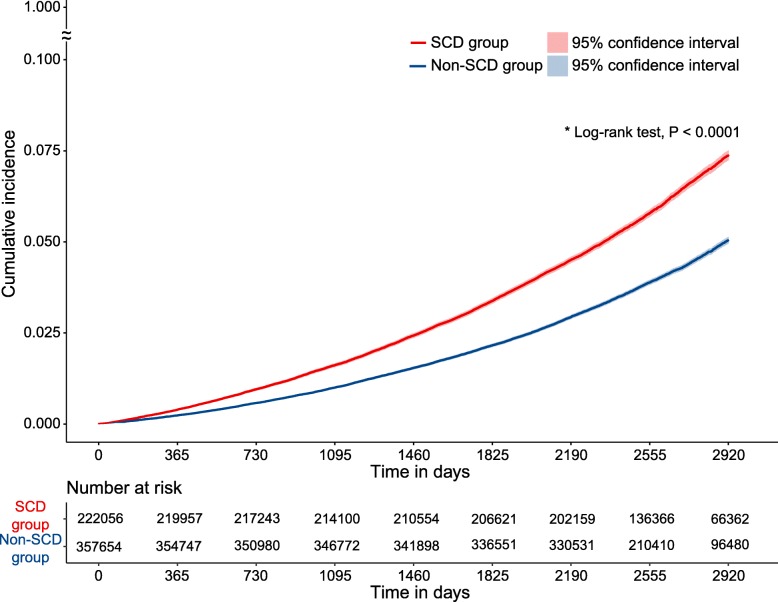
Kaplan-Meier estimates of the incidence of dementia. Cumulative incidence of dementia in non-SCD and SCD groups. SCD, subjective cognitive decline. *A significantly increased risk of dementia was found in the SCD group

Among individuals with SCD, the incidence of dementia was 8.59 per 1000 person-years, which was higher than individuals without SCD who developed dementia at an incidence of 5.66 per 1000 person-years (Table 2). The SCD group had a higher cumulative incidence of dementia compared to the non-SCD group (log-rank *P* < 0.001, Fig. 1). When adjusted for clinical factors (model 3), subjects with SCD had an increased risk of subsequent dementia (aHR = 1.38, 95% CI 1.34 to 1.41 in model 3; see Table 2). The aHRs were consistent in both men and women across all Cox regression models tested, despite controlling for various covariates. The effect of interaction between SCD and sex on subsequent dementia was not significant (Table S3 in the online supplement). The propensity score-matched analysis also confirmed that the presence of SCD increased the risk of subsequent dementia (aHR = 1.39, 95% CI 1.36 to 1.43 in model 3). The incidence of AD and dementia other than AD during the follow-up period is presented in Table S2 in the online supplement. The incidence rates of dementia associated with other risk factors including smoking, alcohol consumption, exercise frequency, depression, cerebrovascular disease, and diabetes mellitus are presented in Table S4 in the online supplement.

**Table 2:** Cox regression analysis for the association between subjective cognitive decline and subsequent dementia

	Non-SCD group	SCD group
Total population	357,654 (61.7%)	222,056 (38.3%)
Dementia events	13,501 (3.8%)	12,766 (5.8%)
Person-years	2,384,745	1,485,548
Incidence (events/1000 person-years)	5.66	8.59
Unadjusted HR (95% CI)	1 [reference]	1.51 (1.47–1.55)
Sex-adjusted HR (95% CI)	1 [reference]	1.48 (1.44–1.51)
aHR in model 1 (95% CI)*	1 [reference]	1.46 (1.43–1.50)
aHR in model 2 (95% CI)^†^	1 [reference]	1.42 (1.39–1.46)
aHR in model 3 (95% CI)^‡^	1 [reference]	1.38 (1.34–1.41)
Men	173,795 (48.6%)	92,516 (41.7%)
Dementia events	5480 (3.2%)	4399 (4.8%)
Person-years	1,147,608	611,069.20
Incidence (events/1000 person-years)	4.78	7.20
Unadjusted HR (95% CI)	1 [reference]	1.50 (1.44–1.56)
aHR in model 1 (95% CI)*	1 [reference]	1.49 (1.43–1.55)
aHR in model 2 (95% CI)^†^	1 [reference]	1.44 (1.39–1.50)
aHR in model 3 (95% CI)^‡^	1 [reference]	1.38 (1.32–1.44)
Women	183,859 (51.4%)	129,540 (58.3%)
Dementia events	8021 (4.4%)	8367 (6.5%)
Person-years	1,237,137	874,478.80
Incidence (events/1000 person-years)	6.48	9.57
Unadjusted HR (95% CI)	1 [reference]	1.47 (1.42–1.51)
aHR in model 1 (95% CI)*	1 [reference]	1.45 (1.40–1.49)
aHR in model 2 (95% CI)^†^	1 [reference]	1.41 (1.37–1.45)
aHR in model 3 (95% CI)^‡^	1 [reference]	1.38 (1.33–1.42)

*aHR* adjusted hazard ratio, *CI* confidence interval, *SCD* subjective cognitive decline

*Adjusted for sex, income, lifestyle factors, and healthcare visit frequency (in subgroup analysis for men and women, sex was not entered as a covariate)

^†^Adjusted for sex, income, lifestyle factors, healthcare visit frequency, medical history, and medication history (in subgroup analysis for men and women, sex was not entered as a covariate)

^‡^Adjusted for sex, income, lifestyle factors, healthcare visit frequency, medical history, medication history, depression screening questionnaire scores, laboratory findings, and physical examination findings (in subgroup analysis for men and women, sex was not entered as a covariate)

### Association between severity of subjective memory impairment and subsequent dementia

**Fig. 2:**
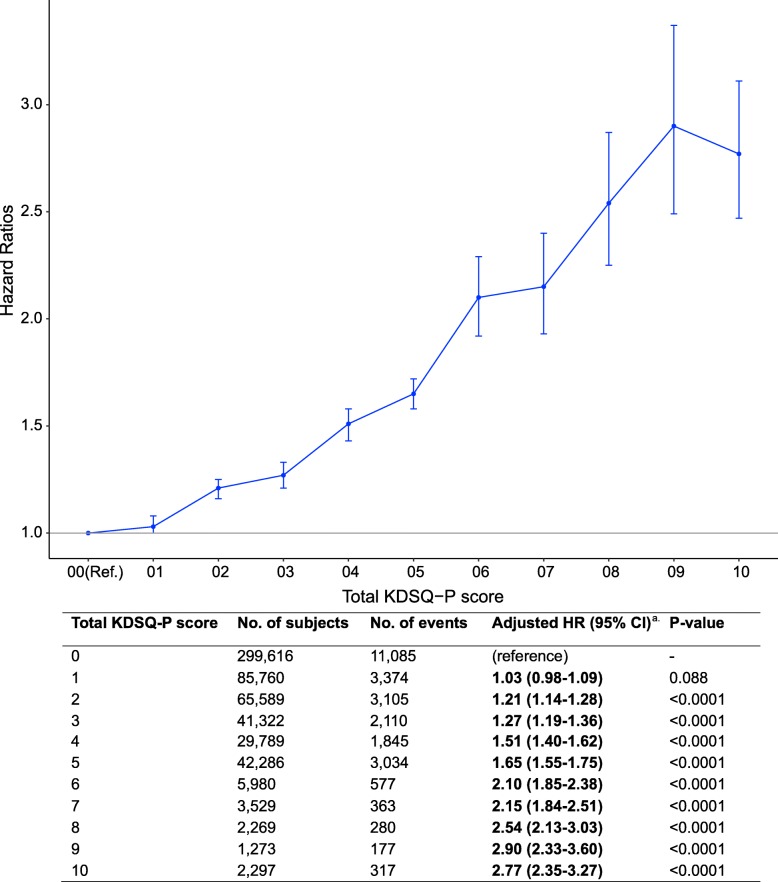
Adjusted HRs for dementia according to the Pre-screening Korean Dementia Screening Questionnaire (KDSQ-P) score. Blue dots indicate the adjusted HR, and blue lines indicate the 95% confidence intervals. HR, hazard ratio; CI, confidence interval; KDSQ-P, Pre-screening Korean Dementia Screening Questionnaire. ^a^Adjusted for sex, income, lifestyle factors, healthcare visit frequency, past medical history, medication history, Depression Screening Questionnaire score, laboratory findings, and physical examination findings

The severity of subjective memory impairment, total KDSQ-P score, was significantly associated with risk of subsequent dementia (Fig. 2). Subjects with a higher KDSQ-P score showed a strong tendency for a higher risk for subsequent dementia. The risk of dementia in subjects with a score of 9 or 10 was approximately three times higher than in subjects with a score of 0. Each item in the KDSQ-P was also significantly associated with the risk of subsequent dementia (see Table S1 in the online supplement). Subjects who answered “frequently yes” for each question had a higher risk for subsequent dementia than those who answered “sometimes yes.”

### Effect of interaction between SCD and depressive symptoms on subsequent dementia

**Fig. 3:**
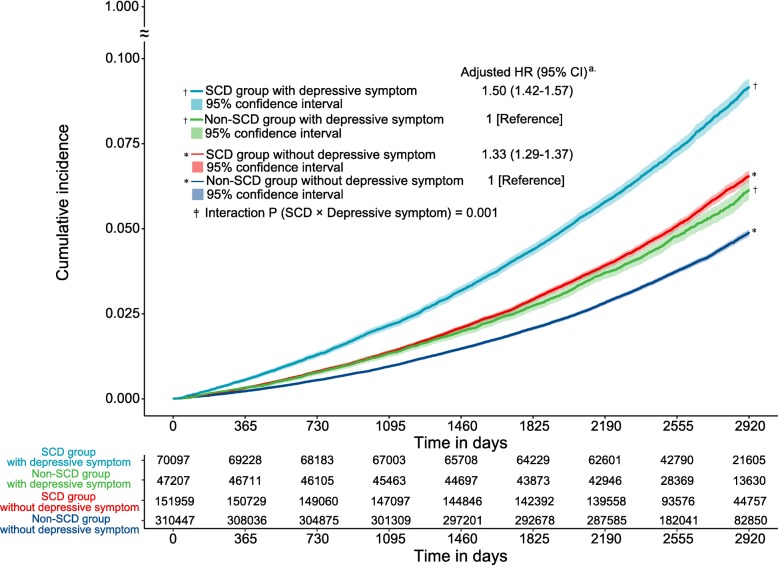
The interaction effect between SCD and depressive symptoms on subsequent dementia. Cumulative incidence of dementia in groups according to the presence of SCD and depressive symptoms. ^‡^A significant interaction was found between SCD and depressive symptoms. SCD, subjective cognitive decline; HR, hazard ratio; CI, confidence interval. ^a^Adjusted for sex, income, lifestyle factors, healthcare visit frequency, past medical history, medication history, laboratory findings, and physical examination findings

Figure 3 shows the estimated effect of SCD on subsequent dementia after accounting for depressive symptoms. Regardless of the presence of depressive symptoms, SCD was significantly associated with a risk for subsequent dementia. Notably, the effect of SCD on subsequent dementia was particularly prominent in the presence of depressive symptoms (in subjects with depressive symptoms, aHR = 1.50, 95% CI 1.42 to 1.57; in subjects without depressive symptoms, aHR = 1.33, 95% CI 1.29 to 1.37; interaction *P* = 0.001).

### Sensitivity analysis for the association between SCD and subsequent dementia

Even after iteratively removing subgroups from our subjects, the risk of subsequent dementia was consistently associated with SCD (see Table S5 in the online supplement). First, we excluded patients diagnosed with subsequent dementia within 1 year of the index date to avoid the onset of actual dementia before reporting SCD. By excluding these patients, our analysis demonstrated the robustness of the link between SCD and subsequent dementia in our study cohort (aHR = 1.37, 95% CI 1.34 to 1.41 in model 3). The results were also consistent when subsequent dementia was subdivided into AD (only including AD, aHR = 1.38, 95% CI 1.35 to 1.42 in model 3) and dementia other than AD (only including dementia other than AD, aHR = 1.37, 95% CI 1.30 to 1.45 in model 3). The definition and the incidence of dementia other than AD are presented in Table S2. We also observed a significant link after excluding individuals with psychiatric disorders (aHR = 1.38, 95% CI 1.34 to 1.43 in model 3), patients with neurological diseases (aHR = 1.43, 95% CI 1.38 to 1.49 in model 3), patients with depressive symptoms according to the DSQ (aHR = 1.33, 95% CI 1.29 to 1.37 in model 3), and patients with KDSQ-P scores ≥ 4 (aHR = 1.18, 95% CI 1.14 to 1.21 in model 3). In addition, the association between the severity of SCD (score 1 or 2 to item 2) and the risk of dementia remained significant after excluding the non-SCD group (score 0 to item 2) (Table S6 in the online supplement).

## Discussion

In this nationwide population-based study of 579,710 66-year-old adults, subjects with SCD were more likely to develop subsequent dementia than those without SCD over an average follow-up period of 6.68 years. The association between SCD and subsequent dementia was robust across sex, subtype of dementia (AD or other than AD), history of psychiatric disorders or neurological diseases, and presence of depressive symptoms. The severity of subjective memory impairment was also associated with the risk of subsequent dementia. Furthermore, regardless of the presence of depressive symptoms, SCD was significantly associated with subsequent dementia, with an increased association in the presence of depressive symptoms.

The positive association between SCD and subsequent dementia found in our study is generally consistent with previous studies. A recent population-based study (*n* = 2710) reported an aHR in SCD similar to that of our study (aHR = 1.18, 95% CI 1.03 to 1.33) [19]. The prevalence of SCD in our study was 38.3% (222,056 in 579,710), which is comparable to the prevalence estimates of previous community-based studies, which ranged from 22.1 to 56.0% [5]. However, the rate of incident dementia and risk of subsequent dementia in the SCD group compared to the non-SCD group in our study was lower than in previous research. In a recent multicenter cohort study of 4369 participants, the incidence rate of dementia in SCD cases was reported to be 17.7 per 1000 person-years [10], which is higher than our result of 8.6 per 1000 person-years. Discrepancies between our results and those of previous SCD studies may be due to the heterogeneity of the study populations [10, 12]. Reports have indicated that, when compared to community populations, patients who visited memory clinics had a higher progression rate from normal cognition to MCI [28], from SCD to AD [10], and from MCI to AD [29].

The higher progression rate observed in memory clinic samples has been attributed to the subjects’ greater likelihood of experiencing the early signs of neurodegenerative diseases and of spontaneously reporting memory complaints [9, 10, 14, 28, 29]. More importantly, decreased functional abilities were found in memory clinic attendees at baseline, which significantly indicates a risk for dementia [28, 29]. In contrast to previous studies, our study consisted of subjects obtained from a population-based setting, and thus better illustrates the robust association between SCD and subsequent dementia in the general population. In accordance with a previous study [19], our analyses revealed equivalent incidence rates of dementia associated with SCD (8.6 per 1000 person-years) and other risk factors including current smoking (7.24/1000 person-years), heavy alcohol drinking (7.44/1000 person-years), no exercise (7.54/1000 person-years), diabetes mellitus (8.91/1000 person-years), cerebrovascular disease (11.34/1000 person-years), and depression (12.46/1000 person-years). This result suggests that SCD has a similar magnitude of risk for subsequent dementia as other lifestyle and vascular risk factors in a community population.

Our analysis showed a higher incidence of dementia in women with SCD than in men with SCD (9.57 vs. 7.20/1000 person-years), but the risk of dementia associated with SCD was comparable in both sexes after adjusting for various factors (aHR = 1.38, 95% CI 1.33 to 1.42 for women; aHR = 1.38, 95% CI 1.32 to 1.44 for men; Table 2 and Table S3 in the online supplement). Some studies reported women to be more susceptible than men to progression from SCD to dementia [30, 31], whereas others found no significant sex difference [8, 10, 19]. Some have reported a tendency for women to report SCD worries with a higher sensitivity to subtle cognitive symptoms relating to dementia progression when compared to men [32]. Women are also known to be susceptible to dementia, possibly due to their longevity and sex-specific biological factors [33]. However, in our study with the largest sample size, before and after adjusting for various clinical factors and sociodemographic variables, the risk of dementia associated with SCD was comparable in both sexes.

Our results also highlight the positive linear association between the severity of subjective memory impairment and subsequent dementia (Fig. 2). This finding suggests that the more severe the subjective memory complaints, the greater the risk of subsequent dementia. KDSQ-P, a validated pre-screening tool for dementia [22], includes items measuring subjective memory using multiple response types. In recent studies, many authors have evaluated SCD with multiple items [34, 35], and some have administered face-to-face interviews [8, 30, 34, 36]. Moreover, many of them asked about specific memory (70.7%) and functional decline (41.6%) to assess SCD [34]. The single question defining SCD in this study lacked information on concerns, non-memory domains, and impairment. However, the use of a general question to identify the presence of SCD and a variety of additional questions regarding specific subjective memory impairment may also clarify the effect of well-defined features of SCD on subsequent dementia.

In this study, the SCD group with depressive symptoms had a greater risk for subsequent dementia than the group without depressive symptoms, with a significant interaction effect (Fig. 3). Although depressive symptoms are regarded as a crucial factor for subsequent dementia due to their association with cognitive disorders [7, 13, 16, 37], previous studies have found a minimal effect of mood scores on the association between SCD and further cognitive decline [8, 19, 38]. This is possibly attributable to the limited size of the studies. Our results imply that SCD and depressive symptoms not only act independently as risk factors for dementia but also contribute to its development through their interaction.

We observed that SCD was likely to be an incipient symptom of both AD and non-AD-related dementias (see Table S5 in the online supplement). Studies have suggested that SCD is related to AD pathology. It has been demonstrated that AD biomarkers such as cerebrospinal fluid β-amyloid [39, 40], plasma β-amyloid [41], hippocampal atrophy [41], and amyloid retention in positron emission tomography [42] are associated with SCD. Although the prevalence of AD pathology in SCD may differ between memory centers due to their varied study designs [43], SCD might be an early symptom in the preclinical stage of AD. Previous studies have reported inconsistent results regarding the association between SCD and non-AD dementia, such as vascular dementia, Lewy body dementia, and frontotemporal lobar degeneration [8, 10]. Although the typical symptoms of dementia differ according to the case, memory dysfunction could represent an early symptom in all forms of dementia [44]. Importantly, memory dysfunction can have diverse manifestations including difficulties with episodic and semantic memory and encoding, retrieval, and recognition types of memory. Our results suggest that SCD can broadly be used as a risk indicator for a myriad of cognitive disorders such as AD and non-AD.

The major strength of our study is that we have used the largest nationwide representative cohort data to date relating SCD to subsequent dementia. We analyzed 579,710 eligible subjects, extracted from over 50 million entries in the NHIS database. Clinical cohorts in SCD research have relatively small to modest numbers of selective participants, ranging from 42 to 4500 [10, 34]. In addition, studies that have assessed the risks associated with subjective memory complaints have used diverse and inconsistent characteristics, including the number of participants (17 to 2901), the age of participants (18 to 87), the follow-up periods (1 to 15 years), the operational criteria for defining SCD, and the methods of assessing dementia [6, 12, 34]. Consequently, when these studies are combined for meta-analysis, the significant heterogeneity between studies may add significant noise towards estimating the association between SCD and dementia. As an additional strength, our results are based on the mandatory national healthcare screening service, which is more reflective of the general population and might be more robust and generalizable than studies conducted through memory clinics. In this study, measuring SCD in a large homogeneous community population with comprehensive information enabled us to investigate SCD and risk for both AD and non-AD dementia with a wide range of clinical covariates, extended time frame, consideration of depressive disorder and subclinical symptoms, and comparison with peers of the same age without SCD.

This study also has several limitations. Firstly, the main weakness is the lack of objective cognition test results. Normal performance on standardized cognitive tests is one of the research criteria for SCD [14]. To reduce bias related to this limitation, we excluded subjects with pre-existing cognitive decline from the analysis, namely subjects with impaired ADLs, a documented history of dementia, MCI, or a prescription for dementia medication. Secondly, although we comprehensively adjusted for various confounds, we did not consider the years of education, occupational attainment, family history, imaging biomarkers, or other potentially relevant confounds. However, we adjusted for covariates such as comprehensive disease diagnosis, income level, and healthcare visit frequency that can only be obtained from the national data. Thirdly, the operational definition of AD may be susceptible to misdiagnosis or underdiagnosis, although the incidence rate of AD in our study population was similar to the rates reported in epidemiological studies conducted in South Korea [45]. Fourthly, the age of 66 years of this cohort is relatively young, and thus, the findings may not represent the entire elderly group. Finally, because the study population included individuals from only a single country, our findings may not be generalizable to people of other backgrounds.

## Conclusion

Our study, in a population-based cohort, is the largest to date and demonstrates the importance of SCD as an early, independent risk factor for dementia. These findings thus provide strong evidence for the role of SCD in characterizing the initial high-risk stage of dementia. As a growing public health issue, SCD should be further investigated as a risk factor for dementia. Giving additional attention to SCD as a risk factor for dementia could facilitate more focused surveillance from the public and healthcare professionals. However, it may not be appropriate for the public to view SCD as a disease state that should be actively treated. Instead, an approach focused on prevention for people with SCD, including lifestyle modifications or providing education on dementia, could be promising. Future studies should further explore the clinical and neurobiological nature of SCD as an early sign of dementia.

## Supplementary information

**Additional file 1.** Supplementary methods, tables, figures, and references.

## Data Availability

This study is based on the National Health Insurance Service (NHIS) register data in South Korea (NHIS-2019-1-211). Because these data belong to the NHIS, the authors are not permitted to share them, except in aggregate (as, for example, in a publication). However, interested parties can obtain the data on which the study was based by submitting a research protocol to the NHIS (https://nhiss.nhis.or.kr/bd/ab/bdaba000eng.do). The analytic/statistical codes are available from the corresponding author (wjmyung@snubh.org, WM), upon reasonable request.

## Abbreviations

AD: Alzheimer’s disease
ADLs: Activities of daily living
aHR: Adjusted hazard ratio
CI: Confidence interval
DSQ: Depression Screening Questionnaire
KDSQ-P: Korean Dementia Screening Questionnaire
MCI: Mild cognitive impairment
NHIS: National Health Insurance Service
NSPTA: National Screening Program for Transitional Ages
SCD: Subjective cognitive decline
