# Supplementary material for: Subjective cognitive decline and subsequent dementia: a nationwide cohort study of 579,710 people aged 66 years in South Korea
Source: Alzheimers Res Ther. 2020 May 6;12:52. doi: 10.1186/s13195-020-00618-1 (PMC7203882; doi:10.1186/s13195-020-00618-1)
Supplement: Supplementary file 1 — Additional file 1. Supplementary methods, tables, figures, and references. [file 13195_2020_618_MOESM1_ESM.docx]

**Subjective cognitive decline and subsequent dementia: a nationwide cohort study of 579,710 people aged 66 years in South Korea**

Yeong Chan Lee,^*^ Jae Myeong Kang,^*^ Hyewon Lee,^*^ Kiwon Kim, Soyeon Kim, Tae Yang Yu, Eun-Mi Lee, Clara Tammy Kim, Doh Kwan Kim, Matthew Lewis, Hong-Hee Won^†^, Frank Jessen, Woojae Myung^†^

**Supplementary Material**

Index of supplementary material

**1. Supplementary methods**……………………………………………………………………………………….3

**2. Supplementary tables**

Table S1: Cox regression analysis for risk of subsequent dementia according to the five items of the Pre-screening Korean Dementia Screening Questionnaire (KDSQ-P)…….…………………………………….4

Table S2: Definition of psychiatric disorders, neurological diseases, medical conditions, and dementia other than Alzheimer’s disease ……………………………………….…………………………………………….…...5

Table S3. Hazard ratios of sex associated with dementia by Cox proportional hazards model…………………….6

Table S3. Incidence rate of dementia associated with other risk factors.………………………….………...…….7

Table S4: Sensitivity analysis: Cox regression analysis for the association between SCD and subsequent dementia………………….…………………………………………………………………………………..8

Table S5. Association between severity of SCD and subsequent dementia after excluding non-SCD group…….9

**3. Supplementary figures**

Figure S1: A flow diagram of the study population …….…………………………………………….....………10

**4. References**………………………………………………………………………….……….…………...…….11

**1. Supplementary methods**

**Data sources and study cohort**

Data were obtained from the South Korean National Health Insurance Service (NHIS) database [1, 2]. The NHIS provides mandatory healthcare for most South Koreans (97%) under a single-payer model. The NHIS collects data on all the healthcare usage of approximately 50 million citizens, including diagnostic codes, medication prescriptions, admission or outpatient visit records, and national health examination data. Since 2007, the NHIS has also provided an age-specific national health examination program, the National Screening Program for Transitional Ages (NSPTA), for all Korean citizens aged 40 or 66 [3]. The program focuses on these age groups to detect diseases in important lifecycle transition periods. For the 66-year-old group, NSPTA records contain information on physical examinations, laboratory tests of urine and blood, questionnaires for cognitive function, depressive symptoms, and functional activities. Our study population consisted of a subset of individuals from the NHIS database who participated in the NSPTA at age 66 between 2009 and 2011. The study population covered 51.8% of the total South Korean population aged 66 during the enrolment period.

**Covariates**

We assessed demographic variables such as sex and income, which we defined from lowest to highest as Medicaid aid, first income group (1^st^ to 6^th^ ventiles), second income group (7^th^ to 14^th^ ventiles), and third income group (15^th^ to 20^th^ ventiles). Lifestyle variables such as smoking status, alcohol consumption habits, and exercise frequency were included as covariates. We adjusted for healthcare visit frequency in the survival analyses. Information on medical history, including psychiatric disorders, neurological diseases, and medical diseases, was defined based on ICD-10 codes before the index date (Table S2). Use of medications was assessed before the index date. We defined a positive prescription history when individuals had received medications for at least 180 days. Information from laboratory tests and physical examinations included low-density lipoprotein cholesterol (mg/dL), high-density lipoprotein cholesterol (mg/dL), triglycerides (mg/dL), fasting glucose (mg/dL), haemoglobin (g/dL), systolic blood pressure (mmHg), diastolic blood pressure (mmHg), and body mass index (weight in kg divided by height in m^2^), all entered as covariates.

We used a depression screening questionnaire (DSQ) to assess depressive symptoms. This questionnaire includes three questions derived from a modified geriatric depression scale [4] (e.g. ‘Have you lost much of your activity or motivation these days?’, ‘Do you feel that you are worthless now?’, and ‘Do you feel that you have no hope now?’). Each question can be self-answered with two possible choices, ‘yes’ or ‘no’, scored as 1 or 0, respectively. Total DSQ scores range from 0 to 3, with higher scores indicating more depressive symptoms.

**Supplementary Table S1. Cox regression analysis for the risk of subsequent dementia according to the five items of the Pre-screening Korean Dementia Screening Questionnaire (KDSQ-P)**

|  | **Comparisons** | **Adjusted HR**  **(95% CI)** ^a^ |
| --- | --- | --- |
| Item 1: ‘Do you think your memory is not as good as your friends’ or colleagues’?’ | ‘0: no’ (reference) | 1 [Reference] |
|  | ‘1: sometimes yes’ or ‘2: frequently yes’ | 1.49 (1.45–1.53) |
|  | ‘0: no’ (reference) | 1 [Reference] |
|  | ‘1: sometimes yes’ | 1.41 (1.38–1.45) |
|  | ‘2: frequently yes’ | 2.24 (2.13–2.37) |
| Item 2: ‘Do you think your memory has declined compared to a year ago?’ | ‘0: no’ (reference) | 1 [Reference] |
|  | ‘1: sometimes yes’ or ‘2: frequently yes’ | 1.38 (1.34–1.41) |
|  | ‘0: no’ (reference) | 1 [Reference] |
|  | ‘1: sometimes yes’ | 1.31 (1.28–1.35) |
|  | ‘2: frequently yes’ | 2.17 (2.06–2.29) |
| Item 3: ‘Are there any cases in which your memory is a problem in doing important things?’ | ‘0: no’ (reference) | 1 [Reference] |
|  | ‘1: sometimes yes’ or ‘2: frequently yes’ | 1.41 (1.37–1.45) |
|  | ‘0: no’ (reference) | 1 [Reference] |
|  | ‘1: sometimes yes’ | 1.34 (1.30–1.38) |
|  | ‘2: frequently yes’ | 2.26 (2.13–2.41) |
| Item 4: ‘Does anyone know that your memory has declined?’ | ‘0: no’ (reference) | 1 [Reference] |
|  | ‘1: sometimes yes’ or ‘2: frequently yes’ | 1.56 (1.51–1.60) |
|  | ‘0: no’ (reference) | 1 [Reference] |
|  | ‘1: sometimes yes’ | 1.50 (1.46–1.55) |
|  | ‘2: frequently yes’ | 2.31 (2.13–2.50) |
| Item 5: ‘Do you think that you have been clumsier than before when doing daily work you used to do well?’ | ‘0: no’ (reference) | 1 [Reference] |
|  | ‘1: sometimes yes’ or ‘2: frequently yes’ | 1.32 (1.29–1.36) |
|  | ‘0: no’ (reference) | 1 [Reference] |
|  | ‘1: sometimes yes’ | 1.27 (1.23–1.31) |
|  | ‘2: frequently yes’ | 2.16 (2.02–2.32) |

Abbreviations: CI, confidence interval; HR, hazard ratio.

^a^ Adjusted for sex, income, lifestyle factors, healthcare visit frequency, psychiatric disorders, neurological diseases, medical diseases, medication history, depression screening questionnaire scores, laboratory findings, and physical examination findings.

Supplementary Table S2. Definition of psychiatric disorders, neurological diseases, medical conditions, and dementia other than Alzheimer’s disease

| **Conditions** | **ICD-10 code** |
| --- | --- |
| **Definition of psychiatric disorders, neurological diseases, and medical conditions** | |
| Psychiatric disorders |  |
| Depression | F32, F33, F34·1 |
| Bipolar affective disorder | F30, F31, F34·0 |
| Substance use disorder | F10–F19 |
| Panic disorder | F41·0 |
| Obsessive-compulsive disorder | F42 |
| Personality disorder | F60, F61 |
| Other psychiatric disorder | F04–09, F34·8, F34·9, F38–F40, F41·1–9, F43–F45, F48, F50, F52-F59, F62–F69, F70–F99 |
| Neurological diseases |  |
| Cerebrovascular disease | G45, G46, I60–I69, H340 |
| Epilepsy | G40, G41 |
| Migraines | G43 |
| Headaches | G44 |
| Sleep disorder | G47, F51 |
| Head injury | S00–S09 |
| Medical diseases |  |
| Diabetes mellitus | E10–E14 |
| Myocardial infarction | I21, I22, I252 |
| Congestive heart failure | I099, I110, I130, I132, I255, I420, I425–I429, I43, I50, P290 |
| Liver disease | B18, K70, K71, K721, K729, K73, K74, K76, I85, I864, I982, Z944 |
| Renal disease | N032–N037, N052–N057, N18, N19, N250, I120, I131, Z490–Z492, Z940, Z992 |
| Peptic ulcer disease | K25–K28 |
| Thyroid gland disorder | E03, E05, E06 |
| Asthma | J45 |
| Cancer | C00–C97 |
| **Definition and incidence of Alzheimer’s disease and dementia other than Alzheimer’s disease during the follow-up period** | |
| Alzheimer’s disease (n=20,914) | F00, G30 |
| Dementia other than Alzheimer’s disease (n=5,353) |  |
| Vascular dementia (n=3,073) |  |
| Vascular dementia | F01 (n=10) |
| Vascular dementia of acute onset | F01·0 (n=52) |
| Multi-infarct dementia | F01·1 (n=107) |
| Subcortical vascular dementia | F01·2 (n=148) |
| Mixed cortical and subcortical vascular dementia | F01·3 (n=144) |
| Other vascular dementia | F01·8 (n=696) |
| Vascular dementia, unspecified | F01·9 (n=1,916) |
| Dementia in other diseases classified elsewhere (n=166) |  |
| Dementia in Pick’s disease | F02·0 (n=7) |
| Dementia in Creutzfeldt–Jakob disease | F02·1 (n=1) |
| Dementia in Parkinson’s disease | F02·3 (n=107) |
| Dementia in other specified disease classified elsewhere | F02·8 (n=51) |
| Unspecified dementia (n=1,867) |  |
| Unspecified dementia | F03 (n=1,867) |
| Other degenerative diseases of the nervous system, not classified elsewhere (n=247) | |
| Behavioural variant of frontotemporal dementia | G31·00 (n=39) |
| Non-fluent primary progressive aphasia | G31·02 (n=3) |
| Other circumscribed brain atrophy | G31·08 (n=39) |
| Senile degeneration of brain, not classified elsewhere | G31·1 (n=38) |
| Degeneration of nervous system due to alcohol | G31·2 (n=1) |
| Lewy body dementia | G31·82 (n=48) |
| Corticobasal syndrome | G31·83 (n=2) |
| Other specified degenerative diseases of the nervous system | G31·88 (n=10) |
| Degenerative disease of the nervous system, unspecified | G31·9 (n=67) |

**Supplementary Table S3.** Hazard ratios of sex associated with dementia by Cox proportional hazards model

|  | **Total population** | **Non-SCD group** | **SCD group** | **Interaction P** |
| --- | --- | --- | --- | --- |
|  | **aHR (95% CI)** ^a.^ | **aHR (95% CI)** ^a.^ | **aHR (95% CI)** ^a.^ |  |
| Sex |  |  |  | 0.359 |
| Male | 1 [Reference] | 1 [Reference] | 1 [Reference] |  |
| Female | 1.14 (1.10-1.18) | 1.15 (1.09-1.21) | 1.13 (1.06-1.20) |  |

^a^ Adjusted for income, lifestyle factors, healthcare visit frequency, medical history, medication history, depression screening questionnaire scores, laboratory findings, and physical examination findings.

Supplementary Table S4. Incidence rate of dementia associated with other risk factors

| Risk factors |  | Incidence rates  (/1,000 person-years) |
| --- | --- | --- |
| SCD | No | 5.66 |
|  | Yes | 8.59 |
| Smoking status | Never smoked | 7.15 |
|  | Ex-smoker | 4.85 |
|  | Current smoker | 7.24 |
| Alcohol consumption | No drinking: rarely | 6.87 |
|  | Light drinking: 3–4 times per week | 5.17 |
|  | Heavy drinking: almost every day | 7.44 |
| Exercise frequency | Exercise | 6.21 |
|  | No exercise | 7.54 |
| Depression | No | 6.29 |
|  | Yes | 12.46 |
| Cerebrovascular disease | No | 6.08 |
|  | Yes | 11.34 |
| Diabetes mellitus | No | 6.03 |
|  | Yes | 8.91 |

**Supplementary Table S5. Sensitivity analysis: Cox regression analysis for the association between SCD and subsequent dementia**

|  | **Sensitivity analyses** | | | | | |
| --- | --- | --- | --- | --- | --- | --- |
|  | **Analysis excluding patient with developed dementia within a year of index date** | **Analysis only including Alzheimer’s disease as an outcome** | **Analysis excluding patient with history of psychiatric disorders** | **Analysis excluding patients with history of neurological diseases** | **Analysis excluding patients with presence of depressive symptoms (DSQ score > 0)** | **Analysis excluding patients with Total KDSQ-P score ≥ 4** |
| Non-SCD group, n  (n excluded) | 356,811  (843 excluded) | 354,846  (2,808 excluded) | 263,241  (94,413 excluded) | 218,944  (138,710 excluded) | 310,447  (47,207 excluded) | 355,761  (1,893 excluded) |
| SCD group, n  (n excluded) | 221,183  (873 excluded) | 219,511  (2,545 excluded) | 153,789  (68,267 excluded) | 126,788  (95,268 excluded) | 151,959  (70,097 excluded) | 136,526  (85,530 excluded) |
| Unadjusted HR (95% CI) | 1.50 (1.46–1.53) | 1.53 (1.48–1.57) | 1.48 (1.43–1.53) | 1.53 (1.47–1.58) | 1.37 (1.33–1.41) | 1.22 (1.19–1.26) |
| Sex adjusted HR (95% CI) | 1.47 (1.43–1.51) | 1.49 (1.45–1.53) | 1.46 (1.41–1.50) | 1.50 (1.45–1.55) | 1.35 (1.31–1.38) | 1.20 (1.17–1.24) |
| aHR in Model 1 (95% CI) ^a^ | 1.45 (1.42–1.49) | 1.47 (1.43–1.51) | 1.45 (1.41–1.50) | 1.50 (1.44–1.55) | 1.35 (1.31–1.39) | 1.21 (1.17–1.24) |
| aHR in Model 2 (95% CI) ^b^ | 1.41 (1.38–1.45) | 1.43 (1.39–1.47) | 1.43 (1.38–1.47) | 1.48 (1.42–1.53) | 1.33 (1.29–1.37) | 1.19 (1.16–1.23) |
| aHR in Model 3 (95% CI) ^c^ | 1.37 (1.34–1.41) | 1.38 (1.35–1.42) | 1.38 (1.34–1.43) | 1.43 (1.38–1.49) | 1.33 (1.29–1.37) | 1.18 (1.14–1.21) |
|  | **Analysis only including patient with developed dementia within a year of index date** | **Analysis only including dementia other than Alzheimer’s disease as an outcome** | **Analysis only including patient with history of psychiatric disorders** | **Analysis only including patients with history of neurological diseases** | **Analysis only including patients with presence of depressive symptoms (DSQ score > 0)** | **Analysis only including patients with Total KDSQ-P score ≥ 4** |
| Non-SCD group, n  (n excluded) | 344,966  (12,658 excluded) | 346,961  (10,693 excluded) | 94,413  (263,241 excluded) | 138,710  (218,944 excluded) | 47,207  (310,447 excluded) | 1,893  (355,761 excluded) |
| SCD group, n  (n excluded) | 210,163  (11,893 excluded) | 211,835  (10,221 excluded) | 68,267  (153,789 excluded) | 95,268  (126,788 excluded) | 70,097  (151,959 excluded) | 85,530  (136,526 excluded) |
| Unadjusted HR (95% CI) | 1.70 (1.55–1.87) | 1.47 (1.39–1.55) | 1.46 (1.41–1.52) | 1.43 (1.38–1.48) | 1.55 (1.47–1.63) | 0.89 (0.76–1.04) |
| Sex adjusted HR (95% CI) | 1.64 (1.49–1.80) | 1.46 (1.38–1.54) | 1.45 (1.39–1.50) | 1.41 (1.36–1.46) | 1.54 (1.47–1.52) | 0.90 (0.76–1.05) |
| aHR in Model 1 (95% CI) ^a^ | 1.61 (1.47–1.70) | 1.45 (1.37–1.53) | 1.44 (1.39–1.50) | 1.40 (1.36–1.45) | 1.53 (1.45–1.61) | 0.89 (0.76–1.04) |
| aHR in Model 2 (95% CI) ^b^ | 1.54 (1.40–1.70) | 1.40 (1.33–1.48) | 1.41 (1.35–1.46) | 1.37 (1.32–1.42) | 1.50 (1.42–1.57) | 0.90 (0.77–1.05) |
| aHR in Model 3 (95% CI) ^c^ | 1.47 (1.34–1.63) | 1.37 (1.30–1.45) | 1.36 (1.31–1.42) | 1.33 (1.28–1.37) | 1.50 (1.43–1.58) | 0.90 (0.77–1.05) |

Abbreviations: SCD, subjective cognitive decline; aHR, adjusted hazard ratio; CI, confidence interval; DSQ, depression screening questionnaire; KDSQ-P, Pre-screening Korean Dementia Screening Questionnaire.

^a^ Adjusted for sex, income, lifestyle factors, and healthcare visit frequency.

^b^ Adjusted for sex, income, lifestyle factors, healthcare visit frequency, medical history, and medication history.

^c^ Adjusted for sex, income, lifestyle factors, healthcare visit frequency, medical history, medication history, depression screening questionnaire scores, laboratory findings, and physical examination findings.

**Supplementary Table S6.** Association between severity of SCD and subsequent dementia after excluding non-SCD group

|  | **Score 1 group**  **(Score of item 2 = 1)** | **Score 2 group**  **(Score of item 2 = 2)** |
| --- | --- | --- |
| Total population | 205,067 (92.3%) | 16,989 (7.7%) |
| Dementia events | 10,982 (5.4%) | 1,784 (10.5%) |
| Person-years | 137,481 | 111,166.8 |
| Incidence (events/1,000 person-years) | 7.99 | 16.05 |
| Unadjusted HR (95% CI) | 1 [Reference] | 2.02 (1.92–2.12) |
| Sex-adjusted HR (95% CI) | 1 [Reference] | 1.97 (1.87–2.07) |
| aHR in Model 1 (95% CI) ^*^ | 1 [Reference] | 1.91 (1.82–2.01) |
| aHR in Model 2 (95% CI) ^†^ | 1 [Reference] | 1.79 (1.70–1.88) |
| aHR in Model 3 (95% CI) ^‡^ | 1 [Reference] | 1.68 (1.59–1.77) |

Abbreviations: SCD, subjective cognitive decline; aHR, adjusted hazard ratio; CI, confidence interval.

^*^ Adjusted for sex, income, lifestyle factors, and healthcare visit frequency (in subgroup analysis for men and women, sex was not entered as a covariate).

^†^ Adjusted for sex, income, lifestyle factors, healthcare visit frequency, medical history, and medication history (in subgroup analysis for men and women, sex was not entered as a covariate).

^‡^ Adjusted for sex, income, lifestyle factors, healthcare visit frequency, medical history, medication history, depression screening questionnaire scores, laboratory findings, and physical examination findings (in subgroup analysis for men and women, sex was not entered as a covariate).

Figure S1. A flow diagram of the study population


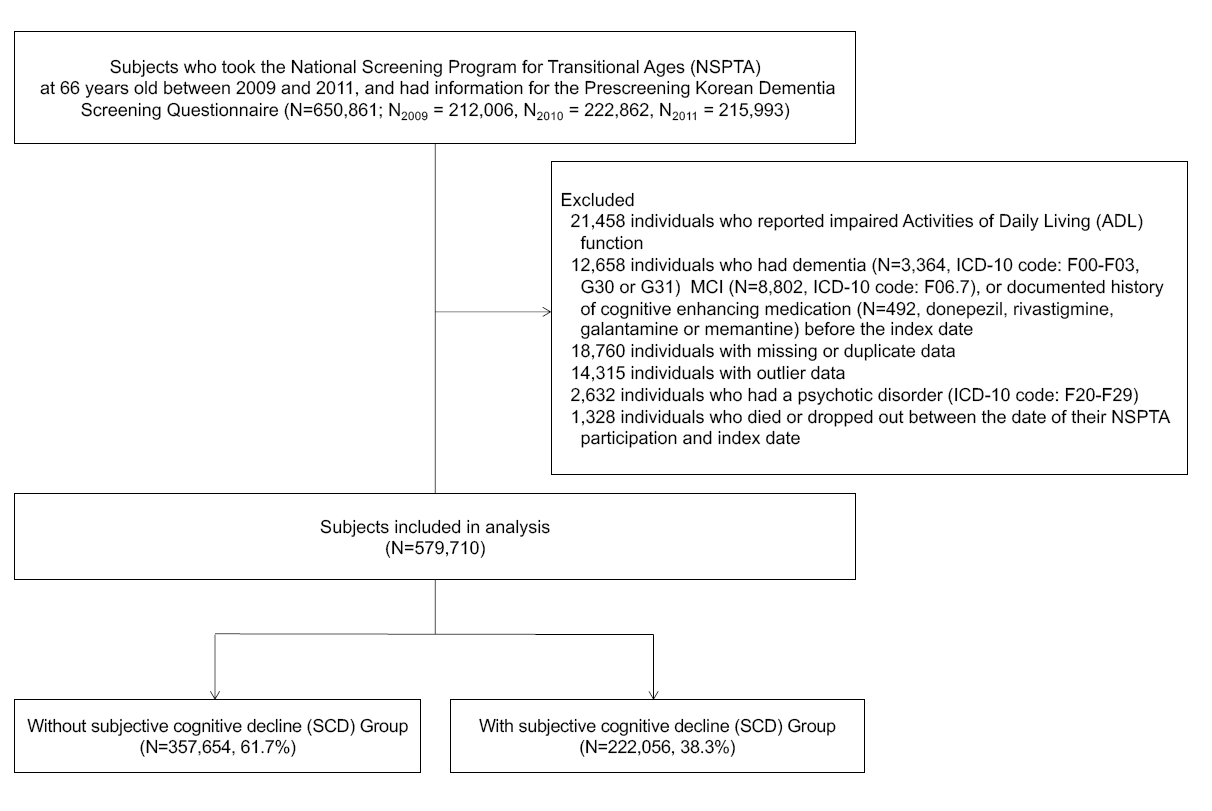


**4. References**

1. Lee J, Lee JS, Park SH, Shin SA, Kim K. Cohort Profile: The National Health Insurance Service-National Sample Cohort (NHIS-NSC), South Korea. Int J Epidemiol. 2017;46:e15.

2. Bahk J, Kim Y-Y, Kang H-Y, Lee J, Kim I, Lee J, et al. Using the National Health Information Database of the National Health Insurance Service in Korea for monitoring mortality and life expectancy at national and local levels. Journal of Korean medical science. 2017;32:1764-70.

3. Kim HS, Shin DW, Lee WC, Kim YT, Cho B. National screening program for transitional ages in Korea: a new screening for strengthening primary prevention and follow-up care. Journal of Korean medical science. 2012;27:S70-S5.

4. Bae JN, Cho MJ. Development of the Korean version of the Geriatric Depression Scale and its short form among elderly psychiatric patients. Journal of psychosomatic research. 2004;57:297-305.
